# The association between anemia and falls in community-living women and men aged 65 years and older from the fifth Tromsø Study 2001-02: a replication study

**DOI:** 10.1186/s12877-017-0689-8

**Published:** 2017-12-27

**Authors:** Laila Arnesdatter Hopstock, Elisabeth Bøe Utne, Alexander Horsch, Tove Skjelbakken

**Affiliations:** 1 Department of Health and Care Sciences, Faculty of Health Sciences, UiT The Arctic University of Norway, N-9037 Tromsø, Norway; 2 Department of Cardiothoracic Surgery, Clinic of Cardiothoracic Surgery, St. Olavs Hospital Trondheim University Hospital, Trondheim, Norway; 3 Department of Computer Science, Faculty of Science and Technology, UiT The Arctic University of Norway, Tromsø, Norway; 4 Division of Internal Medicine, University Hospital North Norway, Tromsø, Norway; 5 Department of Clinical Medicine, UiT The Arctic University of Norway, Tromsø, Norway

**Keywords:** Accidental falls, Anemia, Epidemiology, Frailty, Hemoglobin

## Abstract

**Background:**

Falls are common among elderly people, and the risk increase with age. Falls are associated with both health and social consequences for the patient, and major societal costs. Identification of risk factors should be investigated to prevent falls. Previous studies have shown anemia to be associated with increased risk of falling, but the results are inconsistent. The aim of this study was to investigate the association between anemia and self-reported falls among community-living elderly people. The study is a replication of the study by Thaler-Kall and colleagues from 2014, who studied the association between anemia and self-reported falls among 967 women and men 65 years and older in the KORA-Age study from 2009.

**Methods:**

We included 2441 participants (54% women) 65 years and older from the population-based Tromsø 5 Study 2001-2002. Logistic regression models were used to investigate the association between anemia (hemoglobin <12 g/dL in women and <13 g/dL in men) or hemoglobin level and self-reported falls last year, adjusted for sex, age, medication use and disability. Further, associations between combinations of anemia and frailty or disability, and falls, were investigated.

**Results:**

No statistical significant associations were found between anemia and falls (OR 95% CI: 0.83, 0.50-1.37) or hemoglobin level and falls (OR, 95% CI: 0.94, 0.81-1.09), or with combinations of anemia and frailty or disability, and falls (OR, 95%: CI: 0.94, 0.40-2.22 and 0.78, 0.34-1.81, respectively).

**Conclusions:**

In this replication analysis, in accordance with the results from the original study, no statistically significant association between anemia or hemoglobin and falls was found among community-living women and men aged 65 years or older.

**Electronic supplementary material:**

The online version of this article (10.1186/s12877-017-0689-8) contains supplementary material, which is available to authorized users.

## Background

Falls are one of the major public health problems among elderly people. Among community-living elderly aged 65 and older, 30% experience a fall at least once a year [1, 2]. Falls can result in physical injuries and are associated with higher risk of mortality [3, 4]. Falls can also result in fear for future falls [5], leading to consequences like social isolation. Falls are also associated with high societal costs [6]. Previous studies have found various risk factors for falls in elderly people, including use of medications like sedatives [2, 7, 8], analgesics and antiepileptics [1], urinary incontinence [1], abnormalities in balance and gait, as well as other disabilities [1, 2, 8]. Due to the serious consequences, the identification of modifiable risk factors for falls of elderly people is important.

Anemia, defined by the World Health Organisation (WHO) criteria [9] as a hemoglobin level below 12 g/dL in women and below 13 g/dL in men [9], is common in old age. Anemia has a prevalence of 24% among elderly worldwide [9] and has symptoms including low energy, fatigue, dizziness, and general weakness. In the third National Health and Nutrition Examination Survey (NHANES III) study, the prevalence of anemia was 11% in elderly aged 65 years and older [10]. Similar prevalence has been found in the Longitudinal Ageing Study Amsterdam [11] and in the InChianti Study [12]. Anemia prevalence increases rapidly after the age of 50, approaching a rate of over 20% in elderly aged 85 years or older [10], and has been found to be up to 65% among nursing home residents [8]. In one third of the elderly in the NHANES III, anemia could not be explained by malnutrition or underlying disease [10]. Late life anemia is associated with subsequent physical decline [3], like impaired physical performance not explained by disease [13], increased disability, and muscle weakness [12]. Anemia has found to be an independent risk factor for falls among hospitalized older adults [14], nursing home residents [8, 15], and among community-living elderly when injurious falls [16] or recurrent falls [11] are the outcome. However, there is a need for further studies of the association between anemia and all falls among community-living elderly people.

The association between anemia and falls has been examined in 967 community-living women and men aged 65 and older in the KORA (Cooperative Health Research in the Region of Augsburg)-Age study in 2009, published by Thaler-Kall et al. [17]. KORA-Age is a follow-up study of the four MONICA (Multinational MONItoring of trends and determinants in CArdiovascular disease)/KORA Augsburg Surveys [18] from the region of Augsburg in Germany. An association between anemia and falls was not found in this general population of elderly women and men [17]. These results differ from other studies [11, 16]. In 394 participants from the Longitudinal Ageing Study Amsterdam [11] using fall calendars in a follow-up time of 3 years, anemia was a significant predictor of the outcome recurrent falls. In a study of 47,530 managed care Medicare enrolled patients (709 cases) in the US, anemia was associated with the outcome injurious falls. Differences in methodology may give different results. It is therefore of interest to perform a replication study using the same analysis as in KORA-Age with data from the population based Tromsø Study, which has a design similar to the KORA-Age Study. The objective of the present study is to investigate the relationship between anemia and falls among community-living elderly people, by performing the same analysis in a sample from the Tromsø Study as previously performed in KORA-Age.

## Methods

### The Tromsø Study

The Tromsø Study (9) is an ongoing population-based study in the municipality of Tromsø, North Norway. The study includes seven surveys (Tromsø 1: 1974, Tromsø 2: 1979-80, Tromsø 3: 1986-7, Tromsø 4: 1994-95, Tromsø 5: 2001-02, Tromsø 6: 2007-08 and Tromsø 7: 2015-16) to which total birth cohorts and representative samples were invited (participation rates 65-79%). A total of 45,473 women and men have participated in one or more surveys. Data collection includes questionnaires and interviews, biological sampling, and clinical examinations. The Tromsø Study has been approved by The Regional Committee of Medical and Health Research Ethics (REC North) and the Norwegian Data Protection Authority. The participants have given written informed consent.

### Sample

In 2001-02 a total of 8130 women and men participated in the fifth Tromsø Study (Tromsø 5). Of these, 1885 women and 1594 men were 65 years or older (participation rate 85% and 88%, respectively). The total sample was invited to a first visit including questionnaires and interviews, physical examinations and biological sampling. A subsample attended a second visit including new biological sampling and extended clinical examinations. Eligible for the present analysis were participants aged 65 years and older who 1) answered the fall-question in the interview at the first visit and 2) had a valid hemoglobin measurement from the second visit. We excluded participants with withdrawn consent (*n* = 66), younger than 65 years (*n* = 4619), and participants who had not answered the fall-question at the interview at the first visit and/or were without valid hemoglobin measurement from the second visit (*n* = 1004). Altogether, we included 2441 participants.

### Hemoglobin and fall measurements

Blood samples were collected by standard methods by trained personnel, with the participant sitting. A brief venous stasis applied to the upper arm was released before venipuncture. Hemoglobin was analyzed by an automated blood cell counter (Coulter Counter, Beckman Coulter, Inc., Brea, California) at the Department of Laboratory Medicine, University Hospital of North Norway, Tromsø. As in the KORA-Age study, the hemoglobin level was considered both as a continuous variable and as a dichotomous variable (anemic or non-anemic defined via the WHO criteria [9] as hemoglobin level < 12 g/dL and <13 g/dL, for women and men, respectively). Information on falls were collected with a standard single interview question (*“Did you ever fall last year?”*), by trained personnel. In KORA-Age the fall question was almost identical (*«Did you fall in the previous year?”*), originally derived from the NHANES Study 2003-2004 [19] (*“During the past 12 months, have you had difficulty with falling?”*).

### Covariates

To match the analyses performed in the KORA-Age Study, we included the following variables as covariates: sex, age, hypertension (blood pressure > 140/90 mmHg), medication use (self-reported number of drugs used on a regular basis) and disability (self-reported functional status). Further, we constructed variables of frailty, disability and multimorbidity similar to those in KORA-Age. Frailty was defined by a slight modification of the criteria by Fried et al. [20, 21], i.e. by grip strength (measured by Martins vigorimeter), gait speed (Timed Up and Go (TUG) test), physical activity level (self-reported), exhaustion (self-reported single item from the Hospital Symptom Check List 10 (HSCL-10)). Participants were defined as frail if they met one or more criteria and non-frail if no criteria was met. Only a representative subsample of participants attending the second visit had TUG and grip strength measured, therefore frailty was scored for only a subsample (*n* = 595). Disability was defined by self-reported functional status (difficulties with moving around in the home, getting out of the home without assistance, participate in leisure activities, using public transport, daily shopping), and participants were defined as disabled if they answered yes on one or more of the disability questions. Only participants 70 years and older were given the questionnaire questions on disability, therefore disability could only be scored in a subsample (*n* = 1284). Multimorbidity was defined by a modified version of the Charlson Comorbidity Index (CCI) [22, 23] without weighting (self-reported; yes if ≥3 of the following diseases: asthma/emphysema/chronic bronchitis, cancer (ever), diabetes, stroke, heart attack/angina pectoris, peptic ulcer)). A detailed overview of all variables for the present study is shown in an additional file (see Additional file 1), including definitions of concepts and coherence with the KORA-Age study.

### Statistics

We used means with standard deviations (SD) for continuous variables, and percentages for categorical variables to present descriptive characteristics of the total sample and age groups (Table 1), sex and age groups (Table 2) and fall status (Table 3), as well as for comparison with the KORA-Age sample (Additional file 2). Two-way t-tests for comparisons of means and Chi-square tests for categorical variables were used to compare groups (sex, age, and fall status) (Tables 1, 2 and 3), as well as between Tromsø 5 and KORA-Age (Additional file 2). Further, we used logistic regression models to assess the association between hemoglobin values (as a continuous variable; per SD decrease) or anemia (dichotomous; anemic or non-anemic) and falls (Table 4). Four different models were used; Model 1: unadjusted; Model 2: adjusted for age and sex; Model 3: adjusted for age, sex and medication use; and Model 4: adjusted for age, sex, medication use and disability. To examine the joint effect of anemia and frailty or disability on the occurrence of falls, in a further step, a model combining anemia and frailty, and anemia and disability, was created (Table 5). The participants were classified into four categories: 1) anemic and disabled/frail, 2) anemic and not disabled/frail, 3) not anemic and disabled/frail, and 4) not anemic and not disabled/frail (reference category), in three different models; Model 1: unadjusted, Model 2: adjusted for age and sex, Model 3: adjusted for age, sex and medication use. The results from the logistic regression analyses are presented as odds ratios (ORs) with corresponding 95% confidence intervals (CIs) (Tables 4 and 5). The analyzes were performed in the total sample, in addition to sex- and age-stratified (age-group 65-74 years and 75+ years, in accordance with the KORA-Age Study) analyses, when applicable. No age- or sex-stratification was used for the joint analysis of anemia and frailty/disability due to low subsample sizes. *P*-values < 0.05 were considered statistically significant. All analyses were performed using SPSS 23.0 (IBM Corp., Armonk, NY) and STATA 14 (StataCorp LP, College Station, TX).

**Table 1:** Study sample characteristics by age, mean (SD) or percentages (number of total number measured (n/N). The Tromsø Study 2001-2002

	All			*p*-value^1^
Variable		Age-group	Age-group	
		65-74 years	≥ 75 years	
	(*N* = 2441)	(*N* = 1637)	(*N* = 804)	
Mean age, years	72.0 (4.8)	69.2 (2.9)	77.6 (2.2)	–
Falls^a^, n (%)	33 (803/2441)	32 (518/1637)	36 (285/804)	0.060
Anemia^b^, n (%)	8 (188/2441)	6 (95/1687)	12 (93/804)	< 0.0001
Hemoglobin, g/dl	13.9 (1.1)	13.9 (1.1)	13.7 (1.2)	< 0.0001
Multimorbidity^c^, n (%)	4 (74/1825)	3 (36/1288)	7 (38/537)	< 0.0001
Disability^d^, n (%)	26 (339/1284)	20 (124/635)	33 (215/649)	< 0.0001
Frailty^e^, n (%)	39 (232/592)	25 (16/65)	41 (216/527)	0.011
Hypertension^f^ (%)	65 (1592/2440)	62 (1006/1637)	73 (586/803)	< 0.0001
Use of antihypertensives, n (%)	31 (741/2363)	29 (467/1588)	35 (274/775)	0.003
Use of ≥ 5 drugs^g^, n (%)	16 (293/1841)	15 (180/1196)	18 (113/645)	0.167
Low physical activity, n (%)	8 (180/2147)	7 (98/1471)	12 (82/676)	< 0.0001
Body mass index, kg/m^2^	26.7 (4.1)	26.7 (4.1)	26.6 (4.2)	0.567

^1^P-value for age difference (two-sided t-test for comparisons of means for continuous variables and Chi-square test for categorical variables)

^a^Self-reported fall last year

^b^WHO criteria (< 12 g/dl in women, < 13 g/dl in men)

^c^Self-reported ≥ 3 of the following diseases: asthma/emphysema/chronic bronchitis, cancer (ever), diabetes, stroke, coronary heart disease, peptic ulcer

^d^Self-reported functional status

^e^Modified Fried’s criteria

^f^Blood pressure > 140/90 mmHg

^g^Self-reported drugs used on a regular basis

**Table 2:** Study sample characteristics by sex and age, mean (SD) or percentages (number of total number measured (n/N)). The Tromsø Study 2001-2002

	Women			Men			*p*-value^1^		
Variable	All	Age-group	Age-group	All	Age-group	Age-group	All	Age-group	Age-group
		65-74 years	≥ 75 years		65-74 years	≥ 75 years		65-74 years	≥ 75 years
	(*N* = 1321)	(*N* = 863)	(*N* = 458)	(*N* = 1120)	(*N* = 774)	(*N* = 346)			
Mean age, years	72.3 (4.8)	69.4 (2.9)	77.7 (2.2)	71.8 (4.8)	69.1 (2.9)	77.8 (2.2)	0.013	0.076	0.880
Falls^a^, n (%)	33 (442/1321)	32 (276/863)	36 (166/458)	32 (361/1120)	31 (242/774)	34 (119/346)	0.520	0.756	0.587
Anemia^b^, n (%)	6 (85/1312)	6 (47/863)	8 (38/458)	9 (103/1120)	6 (48/774)	16 (55/346)	0.011	0.514	0.001
Hemoglobin, g/dl	13.4 (1.0)	13.5 (0.9)	13.4 (1.0)	14.5 (1.1)	14.5 (1.1)	14.1 (1.2)	–	–	–
Multimorbidity^c^, n (%)	4 (34/932)	3 (18/659)	6 (16/273)	4 (40/893)	3 (18/629)	8 (22/264)	0.368	0.887	0.264
Disability^d^, n (%)	31 (220/701)	26 (85/334)	37 (135/367)	20 (119/583)	13 (39/301)	28 (80/282)	< 0.001	< 0.0001	0.024
Frailty^e^, n (%)	47 (141/302)	33 (10/30)	48 (131/272)	31 (91/290)	17 (6/35)	33 (85/255)	< 0.001	0.131	0.001
Hypertension^f^ (%)	68 (892/1321)	64 (552/863)	74 (340/458)	63 (700/1119)	59 (454/774)	71 (246/345)	0.010	0.028	0.354
Use of antihypertensives, n (%)	31 (339/1277)	29 (246/839)	35 (153/438)	31 (342/1086)	30 (221/749)	36 (121/337)	0.898	0.935	0.779
Use of ≥ 5 drugs^g^, n (%)	15 (157/1044)	14 (95/663)	16 (62/381)	17 (136/797)	16 (85/533)	19 (51/264)	0.239	0.436	0.317
Low physical activity, n (%)	11 (116/1103)	8 (61/742)	15 (55/361)	6 (64/1044)	5 (37/729)	9 (27/315)	< 0.001	0.016	0.008
Body mass index, kg/m^2^	27.0 (4.5)	26.9 (4.6)	27.0 (4.4)	26.4 (3.5)	26.5 (3.5)	26.1 (3.7)	< 0.001	0.048	0.001

^1^P-value for sex difference (two-sided t-test for comparisons of means for continuous variables and Chi-square test for categorical variables)

^a^Self-reported fall last year

^b^WHO criteria (< 12 g/dl in women, < 13 g/dl in men)

^c^Self-reported ≥ 2 of the following diseases: asthma/emphysema/chronic bronchitis, cancer (ever), diabetes, stroke, coronary heart disease, peptic ulcer

^d^Self-reported functional status

^e^Modified Fried’s criteria

^f^Blood pressure > 140/90 mmHg

^g^Self-reported drugs used on a regular basis

**Table 3:** Fall and non-fall participant characteristics (percentages or means (SD)). The Tromsø Study 2001-2002

	Fall participants			Non-fall participants			*p*-value^1^		
Variabel (n)	All (*N* = 803)	Women (*n* = 442)	Men (*n* = 361)	All (*N* = 1638)	Women (*n* = 879)	Men (*n* = 759)	All	Women	Men
Mean age, years	72.2 (4.9)	72.3 (4.9)	72.1 (4.9)	72.0 (4.8)	72.2 (4.8)	71.6 (4.8)	0.219	0.706	0.172
Mean hemoglobin, g/dl	13.8 (1.1)	13.4 (0.9)	14.4 (1.1)	13.9 (1.1)	13.4 (1.0)	14.4 (1.1)	0.550	0.373	0.739
Anemia^a^, %	7.9	7.0	8.9	7.6	6.1	9.4	0.852	0.543	0.791
Mulitimorbidity^b^, %	4.2	4.6	3.4	4.0	3.2	4.8	0.849	0.332	0.530
Disability^c^, %	28.0	32.0	23.2	25.6	31.0	19.0	0.361	0.795	0.239
Frailty^d^, %	41.3	43.6	38.5	38.1	48.4	27.8	0.450	0.421	0.064
Hypertension^e^, %	63.3	64.7	61.5	66.2	68.9	63.0	0.150	0.121	0.613
Use of antihypertensives, %	33.2	33.7	32.5	30.5	30.0	31.0	0.187	1.277	0.633
Use of ≥ 5 drugs^f^, %	18.1	16.1	20.6	14.9	14.5	15.3	0.077	0.483	0.063
Low physical activity level, %	6.5	8.4	4.5	9.3	11.6	6.9	0.029	0.096	0.126
Body mass index, kg/m^2^	26.8 (4.2)	27.2 (4.6)	26.3 (3.5)	26.7 (4.1)	26.9 (4.5)	26.4 (3.5)	0.471	0.213	0.574

^1^P-value for difference between fall and non-fall participants (two-sided t-test for comparisons of means for continuous variables and Chi-square test for categorical variables)

^a^WHO criteria (< 12 g/dl in women, < 13 g/dl in men)

^b^Self-reported ≥ 2 of the following diseases: asthma/emphysema/chronic bronchitis, cancer (ever), diabetes, stroke, coronary heart disease, peptic ulcer

^c^Self-reported functional status

^d^Modified Fried’s criteria

^e^Blood pressure > 140/90 mmHg

^f^Self-reported drugs used on a regular basis

**Table 4:** Association between hemoglobin or anemia and fall, by sex and age, OR (CI). The Tromsø Study 2001-02

	Unadjusted model	Model 1^b^	Model 2^c^	Model 3^d^
Hemoglobin level (continuous, per SD decrease)				
Total sample	1.03 (0.94-1.12)	1.01 (0.92-1.11)	1.03 (0.93-1.14)	0.94 (0.81-1.09)
Age 65-74	1.04 (0.93-1.15)	1.04 (0.92-1.17)	1.09 (0.95-1.25)	1.06 (0.85-1.32)
Age ≥ 75	0.98 (0.85-1.14)	0.97 (0.83-1.13)	0.94 (0.80-1.11)	0.87 (0.71-1.05)
Women	1.06 (0.93-1.22)	1.06 (0.93-1.22)	1.02 (0.88-1.19)	0.85 (0.62-1.05)
Age 65-74	1.13 (0.94-1.35)		1.09 (0.89-1.34)	0.79 (0.55-1.11)
Age ≥ 75	0.97 (0.79-1.20)		0.94 (0.75-1.18)	0.90 (0.69-1.17)
Men	0.98 (0.86-1.11)	0.97 (0.85-1.10)	1.03 (0.89-1.20)	1.06 (0.87-1.30)
Age 65-74	0.97 (0.82-1.14)		1.09 (0.90-1.31)	**1.40 (1.03-1.91)**
Age ≥ 75	0.97 (0.78-1.20)		0.94 (0.74-1.21)	0.84 (0.63-1.11)
Anemia^a^				
Total sample	1.03 (0.75-1.41)	1.02 (0.74-1.40)	1.00 (0.70-1.44)	0.83 (0.50-1.37)
Age 65-74	1.05 (0.67-1.63)	1.05 (0.68-1.63)	1.06 (0.64-1.77)	1.15 (0.52-2.55)
Age ≥ 75	0.95 (0.60-1.50)	0.96 (0.61-1.52)	0.92 (0.55-1.56)	0.69 (0.36-1.32)
Women	1.15 (0.73-1.82)	1.15 (0.73-1.82)	1.05 (0.62-1.77)	0.55 (0.23-1.29)
Age 65-74	1.10 (0.59-2.05)		0.96 (0.46-2.00)	0.44 (0.09-2.03)
Age ≥ 75	1.16 (0.59-2.29)		1.11 (0.52-2.36)	0.64 (0.22-1.84)
Men	0.94 (0.61-1.46)	0.90 (0.58-1.40)	0.95 (0.57-1.58)	1.08 (0.57-2.03)
Age 65-74	1.00 (0.53-1.88)		1.17 (0.57-2.38)	2.17 (0.76-6.22)
Age ≥ 75	0.83 (0.45-1.54)		0.78 (0.38-1.62)	0.72 (0.32-1.65)

*OR* Odds Ratios, *CI* Confidence Intervals

^a^WHO criteria (<12 g/dl in women, <13 g/dl in men)

^b^Model 1: Adjusted for age (years, only for the total sample and in sex-stratified analyses) and sex (only in the total sample and in the age-stratified analyses)

^c^Model 2: In addition to Model 1 adjusted for number of drugs (self-reported)

^d^Model 3: In addition to Model 2 adjusted for disability (self-reported)

**Table 5:** Associations between anemia^a^ and frailty^b^ or disability^c^, and falls^d^, total sample (n), OR (CI). The Tromsø Study 2001-2002

	Unadjusted model	Model 1^e^	Model 2^f^
Anemic & frail (30)	1.03 (0.48-2.22)	0.98 (0.45-2.13)	0.94 (0.40-2.22)
Anemic, not frail (36)	0.80 (0.38-1.66)	0.77 (0.36-1.61)	0.74 (0.32-1.85)
Not anemic & frail (202)	1.22 (0.91-1.65)	1.15 (0.84-1.59)	1.23 (0.87-1.74)
Not anemic & not frail (2173)	1 (ref)	1 (ref)	1 (ref)
Anemic & disabeled (34)	0.75 (0.35-1.63)	0.74 (0.34-1.60)	0.78 (0.34-1.81)
Anemic, not disabeled (71)	1.00 (0.60-1.68)	0.97 (0.57-1.63)	0.92 (0.50-1.70)
Not anemic & disabeled (305)	1.18 (0.90-1.55)	1.15 (0.87-1.52)	1.14 (0.83-1.56)
Not anemic & not disabeled (874)	1.00 (ref)	1.00 (ref)	1.00 (ref)

*OR* Odds Ratios from logistic regression models, *CI* Confidence Intervals

^a^WHO criteria (< 12 g/dl in women, < 13 g/dl in men)

^b^Modified Fried’s criteria

^c^Self-reported abilities to perform daily activities

^d^Self-reported fall last year

^e^Model 1: Adjusted for age (years) and sex

^f^Model 2: In addition to Model 1 adjusted number of drugs (self-reported)

## Results

### Study sample characteristics

Tables 1 and 2 show the study characteristics in the Tromsø 5 study sample in the total sample and by sex and age. Mean hemoglobin was within normal range; 13.4 g/dL (SD 1.0) in women and 14.4 g/dL (SD 1.9) in men. The prevalence of anemia was 6% in women and 9% in men. The older women had higher mean hemoglobin and lower prevalence of anemia (8%) compared to the older men (16%) (*p* = 0.001). A total of 33% reported to have fallen last year, with no statistically significant sex or age differences. The overall prevalence of multimorbidity, disability and frailty was 4%, 26% and 39%, respectively. Overall, the older age group had lower mean hemoglobin levels and higher prevalence of anemia, multimorbidity, disability and frailty compared to the younger age group. Overall, the women in both age groups had higher prevalence of disability compared to men, and older women had higher prevalence of frailty compared to older men.

### Comparison of the samples from Tromsø 5 and KORA-Age

Comparison between characteristics of the Tromsø 5 Study sample and the KORA-Age Study (Additional file 2) show that mean hemoglobin levels and prevalence of fall was higher in the Tromsø Study, while mean age and body mass index, and prevalence of anemia, multimorbidity, hypertension, polypharmacy and use of antihypertensives was lower, compared to the KORA-Age Study. There was no difference in prevalence of disability and frailty between the two studies.

### Fall and non-fall-participant characteristics

Table 3 show the characteristics of participants who reported falling, compared to participants who reported no fall the last year. There were no statistically significant differences in the characteristics among those who reported falling and those who reported no fall, neither in the total sample or when stratified by sex. The exception was that in the total sample, participants who reported falling had statistically significant higher prevalence of low physical activity level, but not in the sex-stratified groups.

### Association between anemia or hemoglobin and falls

We found no statistically significant associations between anemia or hemoglobin and falls in the logistic regression models (Table 4), neither in the crude analysis, nor when adjusted for sex and/or age (when applicable), drug use and disability (fully adjusted models; OR, 95% CI: 0.83, 0.50-1.37 and 0.94, 0.81-1.09, respectively). The sole exception was a statistically significant association between hemoglobin levels and falls, in the fully adjusted model, in the subsample of men aged 65-74 years (OR, 95% CI per SD decrease 1.40, 1.03-1.91).

### Association between anemia, frailty or disability and falls

We found no statistically significant association between combinations of anemia and frailty, or anemia and disability, and falls (OR 95% CI: 0.94, 0.40-2.22 and 0.73, 0.32-1.67, respectively) (Table 5).

## Discussion

In this replication analysis from the Tromsø Study, in accordance with the main findings from the original study by Thaler-Kall et al. from the KORA-Age Study, we found no statistically significant association between measured anemia or hemoglobin and self-reported falls among women and men aged 65 years and older.

### Comparison of the variables and samples from Tromsø 5 and KORA-Age

Considerable efforts were made to reconstruct the covariate variables from KORA-Age with the available data from the Tromsø Study. However, this was not possible for all concepts.

Both prevalence of anemia and falls were higher in the KORA-Age sample than in the Tromsø Study sample. The prevalence of anemia in men and women was consistent between the current Tromsø 5 Study and a previous analysis from the Tromsø 4 Study (1994-95) [24].

The observed differences in prevalence of anemia, morbidity and medication use between the two samples were likely to be due to the fact that the Tromsø Study participants were on average 4 years younger than the KORA-Age participants. This is not considered to have influenced the differences in main results, as all analyses were performed in either strata of age groups or adjusted for age, in both studies. The prevalence of the covariates for the joint analysis, disability and frailty, did not differ between the studies.

### Fall and non-fall participant characteristics

We did not find differences in characteristics between those who reported falling and those who reported no fall the last year. In Thaler-Kall’s analysis, participants who reported falling last year were older, and had higher prevalence of frailty and disability. Previous studies have reported associations between risk of falls and age [15], frailty [25] and disability [26]. This could not be supported by the current study.

### Association between anemia or hemoglobin and falls

In accordance to the findings from Thaler-Kall et al. [17], we found no independent statistically significant association between hemoglobin or anemia and falls. The exception was an observed association between hemoglobin and falls among men aged 65–74 years, considered a coincidental finding in this subsample, that could not be supported by the analysis of anemia and falls, when adjusted for the same covariates.

Several previous studies including both prospective [11], case-control [14, 27] and retrospective [8, 15, 16] analyses report a positive association between anemia and falls. However, some of these studies investigated only injurious falls as the outcome [16, 27], self-reported anemia as the predictor [27], included selected populations such as hospitalized patients [14] or nursing home residents [8], and/or included smaller study samples [8, 11, 14, 15] than the current study.

### Association between anemia, frailty or disability and falls

We found neither a statistical significant association between combinations of anemia & frailty and falls, nor with combinations of anemia & disability and falls. In the KORA-Age Study [17], a joint effect of anemia & disability and falls was found. Participants who were disabled, with or without anemia, had a higher risk of fall compared to those who were not disabled, with or without anemia. This finding was not supported in the current analysis. In both samples, 25% participants were defined as disabled and 40% as frail. Disability was defined slightly different in the two samples; in the Tromsø Study using a non-validated questionnaire, in KORA-Age by a validated scoring tool for activities of daily living. The definition of frailty was close to identical between the two studies, and no joint effect of anemia & frailty was found in either of the studies. Frailty, disability and comorbidity are distinct clinical entities, however the concepts are often related, and difficult to disentangle [28]. Previous studies have found anemia related to disability [12] and physical function [3]. A recent review study [29] found a U-shaped association between physical activity and falls, where the most inactive and the most active have the highest risk of falling. This can be due to different fall contexts, i.e. an outdoor fall as consequence of for example engaging in sport activities (associated with good health and an active lifestyle) or an indoor fall as a consequence of different impairments (associated with disability, frailty and/or comorbidity). A previous analysis from the Tromsø 4 Study [30] of participants aged 55-75 years, found that men with anemia had a two-fold risk for incident non-vertebral fractures compared to men without anemia, but no association was found between anemia and fractures among women. Risk factors for indoor and outdoor falls differ [31], therefore the context and location of the fall may be a confounder in analyses of predictors of falls in the elderly. Due to limitation of available data, this could not be investigated in the current study.

### Strengths and limitations

A strength of the current analysis is that the design of the Tromsø Study is largely comparable to the KORA-Age Study, consists of a larger study sample, and is therefore suitable for replication purposes. However, the main limitation is that not all covariates used in the KORA-Age analysis were available in the Tromsø Study. Due to differences in available variables and data collection tools, some of the definitions of covariates used in the analysis were not identical between the two studies, which could have led to misclassification bias. Nevertheless, the study designs and variable definitions have been presented in detail, to facilitate comparison and interpretation of results. A general limitation yielding both the current analysis and the KORA-Age analysis is the cross-sectional design that cannot discern possible causal relationships between predictors and outcome. In both studies, there is a gap in time between the measured hemoglobin level and the reported event of falling (up to 1 year), so that the anemia status could have been different at the time of falling. In both studies, the phrasing of the fall question could not distinguish between falls in different contexts, for example whether the fall occurred indoors or outdoors. Further, in The Tromsø Study, the prevalence of anemia in women was two-three times higher when using the WHO criteria compared to the 2.5 percentile [24]. By defining anemia according to the WHO criteria, some non-anemic women were classified as anemic and this could have affected the results in this study. In addition to this, in both The Tromsø Study and the KORA-Age study there is a risk of selection bias, i.e. yielding exclusion of participants who were not able to attend the examinations due to their health status, which could possibly lead to an underestimation of the association between anemia and falls. Nevertheless, the main results of the two studies are coherent, and the high participation rate in the Tromsø Study is a strength for external validity.

## Conclusions

In this replication analysis from the Tromsø Study, in accordance with the results from the KORA-Age study, no statistically significant association between anemia or hemoglobin and falls was found among community-living women and men aged 65 years or older. Further studies using a prospective design to investigate anemia as a predictor for unintentional falls are needed.

## Additional files

Additional file 1: Comparison of variables in The Tromsø 5 Study 2001-01 and the KORA-Age Study 2009. A list of available variables in the Tromsø 5 Study and in the KORA-Age Study used in the analysis, presenting the comparability between the studies. (PDF 211 kb)

Additional file 2: Comparison table of study sample characteristics by sex, mean (SD) or percentages (number of total number measured (n/N). The Tromsø 5 Study 2001-2002 and the KORA-Age Study 2009. Supplementary table of study characteristics in the Tromsø 5 Study and the KORA-Age Study, presenting the comparability between the studies. (PDF 167 kb)

## Abbreviations

CCI: Charlson Comorbidity Index
CI: Confidence interval
HSCL-10: Hospital Symptom Check List 10
KORA: Cooperative Health Research in the Region of Augsburg
MONICA: Multinational MONItoring of trends and determinants in CArdiovascular disease
NHANES: National Health and Nutrition Examination Survey
OR: Odds ratio
SD: Standard deviation
TUG: Timed Up and Go test
WHO: World Health Organisation
