# Supplementary material for: The association between anemia and falls in community-living women and men aged 65 years and older from the fifth Tromsø Study 2001-02: a replication study
Source: BMC Geriatr. 2017 Dec 27;17:292. doi: 10.1186/s12877-017-0689-8 (PMC5745627; doi:10.1186/s12877-017-0689-8)
Supplement: Supplementary file 1 — Comparison of variables in The Tromsø 5 Study 2001-01 and the KORA-Age Study 2009. A list of available variables in the Tromsø 5 Study and in the KORA-Age Study used in the analysis, presenting the comparability between the studies. (PDF 211 kb) [file 12877_2017_689_MOESM1_ESM.pdf]

Additional file 1
Comparison of variables in The Tromsø 5 Study 2001-01 and the KORA-Age Study 2009.

The Tromsø 5 Study 2001-02	The KORA-Age Study 2009
**History of falls**<br>*“Did you ever fall last year?" (“No”*=0, *“Yes”*=1, *“I do not know”*=missing)	**History of falls**<br>*“Did you fall in the previous year?”* (*“No”*=0, *“Yes once”*=1, *“Yes more than once”*=1)
**Hemoglobin** (g/dL)	**Hemoglobin** (g/dL)
**Anemia** (WHO criteria: Women <12 g/dL=1, Men<13 g/dL=1)	**Anemia** (WHO criteria: Women <12 g/dL=1, Men<13 g/dL=1)
**Age** (years, age group 65-74 year and 75+ years)	**Age** (years, age group 65-74 year and 75+ years)
**Sex**	**Sex**
**Frailty** (modified Fried’s criteria) ≥1 of the following:	**Frailty** (modified Fried’s criteria) ≥1 of the following:
	Weight loss last 6 months (short German SCREEN II Questionnaire)
Grip strength (Martins vigorimeter, ≤20% weakest)	Grip strength low (JAMAR dynamometer, cut-off not reported)
Timed Up and Go Test (≥15 seconds=1)	Timed Up and Go Test low (cut-off not reported)
Physical activity level (self-reported)<br>*“How has your physical activity in leisure time been during this last year? Think of your weekly average for the year”* (hours per week).<br>Light physical activity (not sweating or out of breath).<br>(*“None”*=0, *“Less than 1”*=1, *“1-2”*=1<br>*“3 or more”*=1)<br>Hard physical activity (sweating/out of breath).<br>(*“None”*=0, *“Less than 1”*=1, *“1-2”*=1<br>*“3 or more”*=1)	Physical activity level low (Physical Activity Scale for the Elderly, cut-off not reported)
Exhaustion (single item, the Hospital Symptom Checklist-10)<br>*Have you experienced any of the following the last week: “That everything is a struggle?”*<br>(*“No complaint” or “Little complaint”* =0, *“Pretty much”, “Very much”*=1)	Fatigue (self-reported, questionnaire/phrasing cut-off not reported)
**Disability** (self-reported)<br>Due to chronic health problems;<br>*“Is it difficult to move around in your home?”*<br>*“Is it difficult to get out of your home by yourself?”*<br>*“Is it difficult to participate in organizations or other leisure time activities?”*<br>*“Is it difficult to use public transport?”*<br>*“Is it difficult to perform daily shopping?”*<br>(*“No difficulties”*=0, *“Some difficulties”*=1, *“Great difficulties”*=1)	**Disability** (Health Assessment Questionnaire Disability index)<br>Dressing, Rising, Eating, Walking, Hygiene, Reach, Grip, Usual activities (cut-off not reported)
**Comorbidity** (modified Charlson Comorbidity Index, i.e. number of diseases without weighting, self-reported)<br>≥ 3 of the following diseases=1:<br>asthma/emphysema/chronic bronchitis, cancer (ever), diabetes, stroke, heart attack/angina pectoris, peptic ulcer	**Comorbidity** (modified Charlson Comorbidity Index, i.e number of diseases, self-reported)<br>. ≥3 of the following diseases =1:<br>asthma/emphysema/chronic bronchitis, cancer (past 3 years), diabetes, stroke, heart trouble, digestive problems, arthritis/rheumatism, HIV/AIDS, kidney disease, liver problems
**Hypertension** (blood pressure >140/90 mmHg)	**Hypertension** (cut-off not reported)
**Number of drug used** on a regular basis (self-reported)	**Numbers of prescribed drugs** used last 7 days (self- reporte
