# Supplementary material for: The association between anemia and falls in community-living women and men aged 65 years and older from the fifth Tromsø Study 2001-02: a replication study
Source: BMC Geriatr. 2017 Dec 27;17:292. doi: 10.1186/s12877-017-0689-8 (PMC5745627; doi:10.1186/s12877-017-0689-8)
Supplement: Supplementary file 2 — Comparison table of study sample characteristics by sex, mean (SD) or percentages (number of total number measured (n/N). The Tromsø 5 Study 2001-2002 and the KORA-Age Study 2009. Supplementary table of study characteristics in the Tromsø 5 Study and the KORA-Age Study, presenting the comparability between the studies. (PDF 167 kb) [file 12877_2017_689_MOESM2_ESM.pdf]

Additional file 2

Comparison table of study sample characteristics by sex, mean (SD) or percentages (number of total number measured (n/N).
The Tromsø 5 Study 2001-2002 and the KORA-Age Study 2009.

Variable	Tromsø 5 All (N=2441)	KORA-Age All (N=967)	Tromsø 5 Women (n=1321)	KORA-Age Women (n=477)	Tromsø 5 Men (n=1120)	KORA-Age Men (n=490)	p-value[1]
Mean age, years	72.0 (4.8)	76.0 (6.5)	72.3 (4.8)	76.0 (6.6)	71.8 (4.8)	75.9 (6.4)	<0.0001
Falls[2], n (%)	33 (803/2441)	14 (143/967)	33 (442/1321)	19 (91/477)	32 (361/1120)	11 (52/490)	<0.0001
Anemia[3], n (%)	8 (188/2441)	18 (171/967)	6 (85/1312)	16 (75/477)	9 (103/1120)	20 (96/490)	<0.0001
Hemoglobin, g/dl	-	-	13.4 (1.0)	13.0 (1.0)	14.4 (1.1)	14.0 (1.3)	<0.0001
Multimorbidity[4], n (%)	4 (74/1825)	9 (82/967)	4 (34/932)	8 (38/477)	4 (40/893)	9 (44/490)	<0.0001
Disability[5], n (%)	26 (339/1284)	25 (238/967)	31 (220/701)	31 (147/477)	20 (119/583)	19 (91/490)	0.333
Frailty[6], n (%)	39 (232/592)	42 (401/967)	47 (141/302)	41 (196/477)	31 (91/290)	42 (205/490)	0.367
Hypertension[7] (%)	65 (1592/2440)	75 (726)	68 (892/1321)	75 (359/477)	63 (700/1119)	75 (367/490)	<0.0001
Use of antihypertensives[8], n (%)	31 (741/2363)	69 (664/967)	31 (339/1277)	69 (328/477)	31 (342/1086)	69 (336/490)	<0.0001
Use of ≥5 drugs[8], n (%)	16 (293/1841)	32 (305/967)	15 (157/1044)	31 (149/477)	17 (136/797)	32 (156/490)	<0.0001
Body mass index, kg/m^2[8]	26.7 (4.1)	28.4 (4.2)	27.0 (4.5)	28.4 (4.6)	26.4 (3.5)	28.4 (3.9)	<0.0001

[1]P-value for study sample difference, total sample (two-sided t-test for comparisons of means for continuous variables and Chi-square test for categorical variables)
[2]Self-reported fall last year
[3]WHO criteria (<12 g/dl in women, <13g/dl in men)
[4]Tromsø 5: Modified Charlson-Comorbidity Index (self-reported ≥3 of the following: asthma/emphysema/chronic bronchitis, cancer (ever), diabetes, stroke, coronary heart disease, peptic ulcer)
[4]KORA-Age: Modified Charlson-Comorbidity-Index (self-reported ≥3 diseases)
[5]Tromsø 5: Self-reported functional status (moving around in the home, getting out of the home, participate in leisure activities, using public transport, daily shopping)
[5]KORA-Age: Self-reported functional status by the Stanford Health Assessment Questionnaire (dressing, arising, eating, walking, hygiene, reach, grip, activities)
[6]Modified Fried's criteria (low walking speed, low grip strength, exhaustion, low physical activity level (Tromsø 5 and KORA-Age) and weight loss (KORA-Age only)).
[7]Tromsø 5: Blood pressure >140/90mmHg
[7]KORA-Age: Unknown definition
[8]Tromsø 5: Self-reported drugs used on a regular basis
[8]KORA-Age: Self-reported drug intake last 7 days of prescribed drugs used on a regular basis
